# The Extent to Which Menstruation-Related Issues Are Included in Graduate-Level Public Health Curricula

**DOI:** 10.3389/fpubh.2020.00442

**Published:** 2020-08-28

**Authors:** Marni Sommer, Christina Lee, Danting Liu, Caitlin Gruer

**Affiliations:** Department of Sociomedical Sciences, Mailman School of Public Health, Columbia University, New York, NY, United States

**Keywords:** menstruation, public health education, adolescent health, higher education, menstrual hygiene management

## Abstract

**Objectives:** Menstruation is increasingly recognized as an issue in domestic and global public health. Public health graduates of U.S. schools of public health must have adequate competencies to address menstruation and its implications for health and well-being in their future endeavors in research, practice and policy. This study sought to understand the extent to which U.S. schools currently integrate menstruation-related content (menstrual health, menstrual hygiene, etc.) and related competencies into their curricula.

**Methods:** We reviewed the course directories of the top 20 US schools of public health as ranked in 2018. Courses were selected based on inclusion of menstruation and adolescent health-related search terms. Syllabi were subsequently obtained and analyzed for inclusion of specific menstruation-related terms. Syllabi including these terms were further analyzed to determine the level of inclusion of menstruation-related topics in relation to public health competencies, and the area of specialization.

**Results:** Of an estimated 5,000 courses assessed, 28 included menstruation-related topics. Most frequently, this inclusion was minimal (e.g., a single reading or assignment), and was limited in scope. Content was typically found within global health, environmental health, and maternal and child health.

**Conclusions:** Given growing attention to menstruation domestically and globally, and the limited current inclusion of this issue in US schools of public health curricula, graduates may not be receiving adequate training on a critically important topic of relevance within population health. Schools should consider reviewing their curricula to assess whether there are opportunities to integrate menstruation-related content in relation to the relevant public health competencies.

## Introduction

To best prepare future public health practitioners, researchers and policy makers, US schools of public health must provide graduate level training that evolves to address emerging key issues influencing population health. This includes the expanding field surrounding the issue of menstruation, referred to by varying actors as “menstrual hygiene management,” “menstrual health,” “period poverty,” and “menstrual equity,” and the array of areas in which menstrual onset and the menstrual cycle relate to child development and reproductive health more broadly. Graduates from schools of public health pursue careers as practitioners in the public and private sector, and in a range of positions influencing domestic and global population health. To assure that schools are equipping new graduates with the essential competencies for tackling emerging critical issues, it is useful to examine graduate level curricula for select issues as they gain importance. This paper presents the findings from one such review, an analysis that examined US graduate level public health curricula for inclusion of topical content pertaining to menstruation in relation to both domestic and global health.

Menstruation has gained global attention in recent years as a critical under-addressed issue within public health (1–3). A growing body of evidence, primarily from low resource countries, has documented the importance of menstruation in relation to population health, particularly within sexual and reproductive health, gender, education, and water and sanitation (1, 4–7). This includes increasing recognition that girls, women and all individuals who menstruate have specific menstruation-related needs that often go unmet (8, 9), including access to safe toilets with water and disposal (7, 10), affordable sanitary products, and accurate menstruation information (4, 9, 11). Furthermore, as explored in numerous studies, many girls report experiencing shame, fear, and anxiety around menstruation (1, 6, 12), and in some contexts girls and women face menstrual restrictions, taboos and discomfort that limit their actions and mobility, and impact their education (13), health, and work productivity (14, 15).

To date, much attention has focused on the menstruation-related challenges facing schoolgirls in low- and middle-income countries (11, 16), which has led to the development of menstrual health and hygiene policies in a number of countries. There is also growing programming and evidence being generated on the menstrual management-related challenges experienced by the 30 million girls and women displaced around the world (17, 18). However, more evidence is needed on these topics and on the menstruation-related issues impacting numerous other populations, such as women in the workplace or those living in crowded urban slums. Evidence is also needed on the implications of heavy bleeding for girls and women with poor access to healthcare, water and sanitation. Fortunately, awareness is growing for the importance of exploring the critical ways in which menstruation is central to sexual and reproductive health outcomes, including child marriage, vulnerability to anemia, and family planning.

Within the US, consideration of menstruation as a public health issue has been limited. In the 1980's and 1990's, US researchers within the psychology and child development spheres examined the experience of menarche among primarily middle-class white girls (19). More recent research has documented the ethnic, racial, and socioeconomic influences on age of menarche (20), and how early age of menarche increases girls' vulnerability to depression, substance use, earlier sexual initiation and school dropout (21–23). However, there has been minimal recent public health research on girls' experiences of menarche and menstruation across the US (24), and the challenges that low-income girls in particular face managing their menstruation in school and daily life. Most menstruation research has been clinical in nature, conducted among older populations, and focusing on fibroids, endometriosis, and other reproductive disorders (14, 25).

In recent years, a growing advocacy movement has led to a number of policies being enacted in the US and other high-income countries around “menstrual equity” and “period poverty.” Although minimal empirical evidence exists, such efforts seek to address the perceived challenges among vulnerable US populations regarding access to menstrual products (26) and menstruation education (27). While current initiatives focus primarily on low-income girls in school (28), the homeless (29) and the incarcerated (30), the discussion is slowly broadening to include challenges faced by all people who menstruate. Key questions remain in the evidence about the specifics and extent of the menstrual-related challenges faced within these populations, along with a lack of evidence on what effective programming and policy on this complex but important topic should include in the US and other high-income countries.

There is a critical need to assure that today's public health students, whether they become practitioners, researchers, or policymakers, working globally or domestically, are cognizant of the many ways in which menstruation is a fundamental public health issue (11). Graduates must be well-trained on the sensitivities of exploring this issue and its sociocultural complexities, and proficient with the requisite methodological and epistemological tools to identify and investigate the key research questions relating to menstruation within population health, and to implement relevant programming and policy. The study described in this paper provides a starting point for understanding student preparedness for addressing this issue.

## Methods

**Figure 1:**
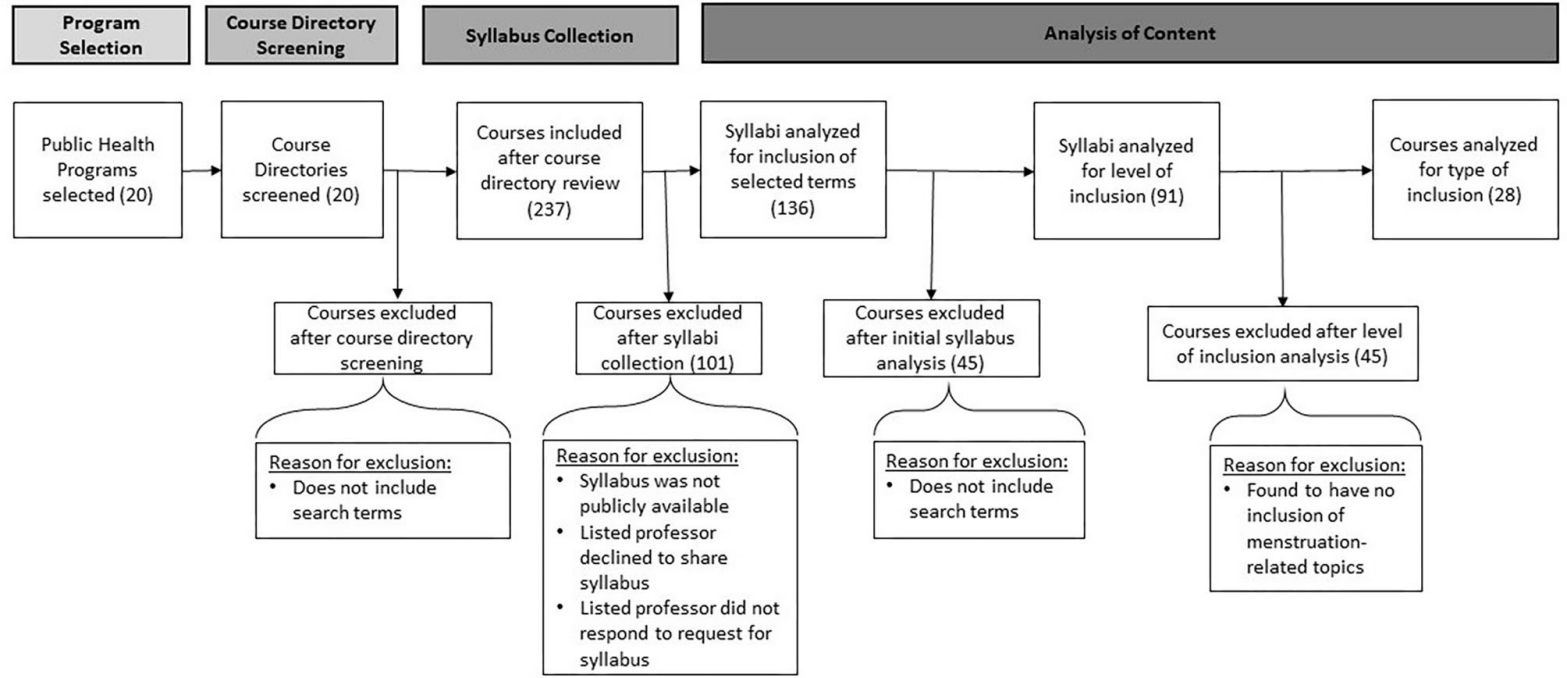
Flow Diagram of Curriculum review and inclusion/exclusion process.

The primary aim of the study was to analyze the extent to which menstruation-related issues are currently included within the graduate-level curricula of schools of public health in the US. The framework was descriptive, exploring whether and how schools of public health have integrated menstruation-related topics into their curricula, through a review of the course directories and select syllabi of the top 20 US schools of public health (Figure 1).

### Selection of Schools of Public Health

The researchers examined the course directories of the top 20 schools of public health in the US, as ranked in the 2018 US News and World Report Best Public Health Schools (Table 1). All are members of Association of Schools & Programs of Public Health (ASPPH) and accredited by the Council of Education for Public Health (CEPH).

**Table 1:** Level of Inclusion of menstruation-related topics in public health courses.

**Level of inclusion**	**Definition**	**# of courses**
Full course	All course sessions are focused on menstruation-related topics	0
Full module	Two or more class sessions are focused on menstruation-related topics	1
Class session	One day or course period is focused on menstruation-related topics	5
Reading	One in-class or out-of-class reading	6
Assignment	One graded task or piece of work assigned to the students focuses on menstruation-related topics	2
Uncategorizable	Incorporate menstrual-related issues in some manner not categorizable in the available categories	14
None	No discernable inclusion of menstruation-related topics	63
**Total**		**91**
Type of Inclusion for Courses categorized as “uncategorizable”:		
• Class includes a lecture^*^ on “Women's sexual and reproductive health”		
• Class includes a lecture^*^ on “pregnancy and reproductive development,” including lecture slides on “puberty”		
• Class includes discussions of puberty for both boys and girls, and the lack of education (formal and informal) for youth about puberty and the changes that come along with it. Discussions are primarily from a biological perspective, and may not necessarily include menstruation		
• Class includes a lecture^*^ on “adolescent development”: puberty, gender identity and social development		
• Class includes a lecture^*^ on “Transitioning to adolescence and young adulthood,” and a reading on identity development in transgender youth		
• Class includes a half session on puberty more broadly		
• Not found in syllabus, but professor noted that menstrual hygiene is referenced in several sessions		
• Class activity on identifying key adolescent health problems linked to development in low- and middle-income countries and vulnerable populations. May include menstruation/menstrual hygiene if selected by students		
• Not found in syllabus, but professor noted that menstrual hygiene is frequently brought up by students, particularly in WASH-related courses		
• Not found in current syllabus, but professor noted in correspondence that students frequently self-select topics related to menstrual hygiene for one of the class assignments. Also, in a different version of the syllabus (readings rotate) included a reading on menstruation		

* *For the purposes of this paper, a lecture is defined as a presentation or talk that does not constitute the entire class period*.

The following public health schools were included in the curricular review: Johns Hopkins Bloomberg SPH; Harvard Chan SPH; University of North Carolina Gillings SPH; University of Michigan, Ann Harbor SPH; Columbia University, Mailman SPH; University of Washington SPH; Emory University, Rollins SPH; University of Minnesota SPH; University of California Berkeley, SPH; Boston University SPH; University of California Los Angeles (UCLA) Fielding SPH; Tulane University, School of Public Health and Tropical Medicine; University of Pittsburgh, SPH; George Washington University, Milken Institute SPH; Yale University, SPH; University of South Florida, College of Public Health; University of Illinois at Chicago, SPH; University of Iowa, College of Public Health; Ohio State University, College of Public Health; and University of Alabama Birmingham, SPH.

### Course Directory Screening

Course listings were accessed through the website of the selected schools. All courses listed in the publicly available course directories were assessed for inclusion. It is estimated over 5,000 courses were reviewed; however, the exact number cannot be provided as the format of some course directories allowed searches to be conducted but did not provide a full list of all courses.

To be selected for review, courses had to have been taught within the last 3 years, with their titles or course descriptions including one or more of the following general search terms (Set 1): “menstruation,” “menses,” “menarche,” “menstrual,” “reproductive health,” “girls' health,” “women's health,” “adolescent health,” “adolescent development,” “global health,” or “sanitation.” Using these search terms, a total of 237 courses were identified.

### Syllabus Collection

The research team sought to obtain syllabi for each of the 237 selected courses. Publicly available syllabi were downloaded. When not publicly available, the research team contacted the listed faculty member to request the syllabus. When no faculty member was recorded, the school registrar or department coordinator was contacted to identify the corresponding faculty member, and then that member was contacted directly. Each faculty member was emailed twice (initial request and follow-up if no response). A total of 136 syllabi were collected for analysis.

### Analysis of Course Content

Two researchers analyzed each of the 136 syllabi to assess the level of inclusion of menstruation and/or menstruation-related issues in the course content.

### Level of Inclusion

Each syllabus was first analyzed for the inclusion of a second, more specific set of menstrual search terms (Set 2): “menstruation,” “menstrual health,” “menstrual hygiene,” “menstrual hygiene management,” “menarche,” “menses,” “puberty,” and “adolescent development.” These terms are the most common phrases and areas of study within existing literature on menstruation-related topics.

Syllabi that included any of these terms were subsequently analyzed for the degree to which the course itself incorporated menstruation-related issues. Categories of analysis were defined as: 1) Full course; 2) Course module; 3) Class Session; 4) Reading; 5) Assignment; 6) Other; and 7) None. Two research team members used a standardized template to categorize the courses, discussing inconsistencies or areas of ambivalence. A third reviewer was consulted to resolve differences.

### Topical Focus

Courses with some level of inclusion of menstrual content—any level besides “None”—were further analyzed to determine the type of content included. Categories of analysis were defined using the ASPPH Common Areas of Study: *Behavioral and Social Science; Biostatistics and Informatics; Community Health; Environmental Health; Epidemiology; Global Health; Health Policy and Management; Health Promotion and Communication; Maternal and Child Health; and Minority Health and Health Disparities*. These common areas of specialization are reflected in the departments and areas of concentration of many of the schools of public health included in this review.

Two additional categories were utilized, *Cross-listed* and *Other*, for courses that did not fall cleanly into an ASPPH Area. Categorization by these topic areas was determined in first instance by the listed host department, or if the department was not included, based on the course content. Given the complementary nature of these topical foci, it is possible that there is some overlap in terms of content between topical focus areas.

## Results

Of the 136 syllabi obtained and analyzed, 91 were determined to potentially have some level of inclusion of menstruation-related content based on the appearance of at least one of the menstrual search terms (Set 2). Of the 91 syllabi including menstruation-related phrases, 28 courses across 14 schools were found to have some inclusion of menstruation-related issues. There was no full course identified that was solely dedicated to menstruation or menstruation-related issues. Only one course (*n* = 1) was found to include a full module on menstruation-related issues. A few courses (*n* = 5) dedicated one class session to the subject, and some additional courses (*n* = 6) included at least one reading on the subject. Two courses included a related assignment (*n* = 2). There were some courses (*n* = 14) that incorporated menstruation-related issues into their coursework in some manner not categorizable in the available categories, such as classes with a related group discussion, or where a component of a lecture was dedicated to the topic. The remaining courses (*n* = 63) had no discernable inclusion of menstruation, or menstruation-related content. The content varied for the readings, lectures and assignments, ranging from providing background on the topic, to coursework encouraging the application of research analysis or program assessment skills in relation to menstruation issues.

**Figure 2:**
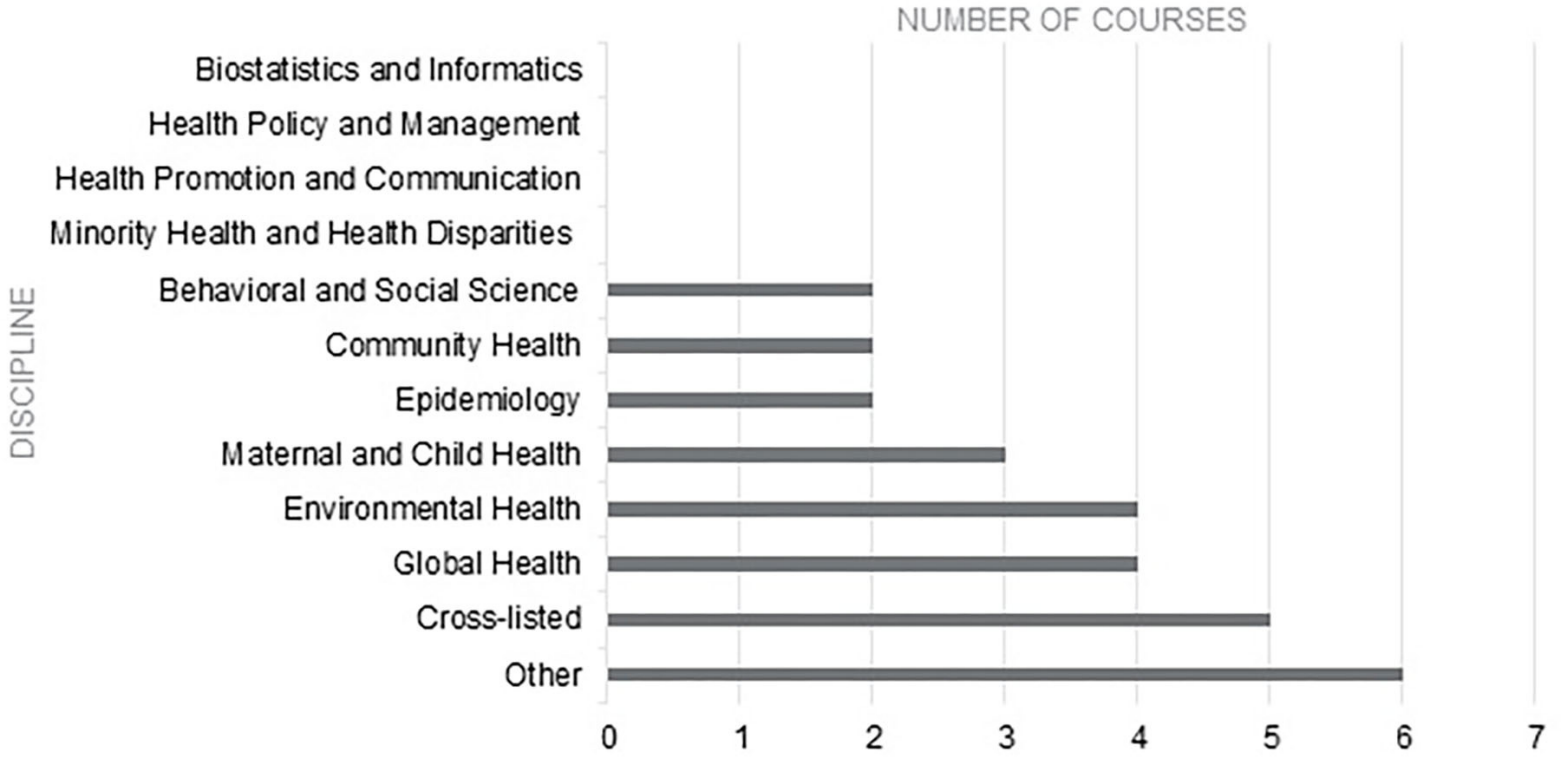
Number of Courses with menstruation related content by discipline.

The courses that included some content relating to menstruation-related topics (*n* = 28) were also identified by area of specialization (See Figure 2). Several courses (*n* = 6) were categorized as “Other,” indicating that the school did not categorize the course by department, or that the course was interdisciplinary. Several courses (*n* = 5) were “Cross-listed” as they are offered across two or more departments or programs. Eight classes with menstruation content were either in Global Health (*n* = 4) or Environmental Health (*n* = 4), with three of the latter focusing primarily on Water & Health or Water, Sanitation, and Hygiene (WASH). These tended to use the challenges girls and women face around menstruation to illustrate the necessity of understanding the distinct and varied needs of a population when designing, implementing, or evaluating an intervention or research project. The remainder of the courses were in Maternal and Child Health (*n* = 3), Behavioral and Social Science (*n* = 2), Community Health (*n* = 2), or Epidemiology (*n* = 2). These inclusions primarily focused on the biological aspects of menstruation such as pubertal timing and the implications of menarche for adolescent- and sexual and reproductive health. No classes were identified in biostatistics and informatics, health policy and management, health promotion and communication, or minority health and health disparities areas of study.

As shown in Table 2, the level of inclusion varied by area of public health specialization. The only course including a full module (*n* = 1) with content relating to menstrual hygiene was in Environmental Health. This module focused on identifying the specific WASH-related challenges that girls face in school, and how to design, implement, and evaluate school-level interventions that address those needs. Courses with at least one class session (*n* = 5) were found across areas, with two in Environmental health, one each in Global Health and Community Health, and one as a cross-listed course. Three of these five courses focused on WASH, with the emphases varying from research methods, to humanitarian emergencies, to designing gender-sensitive WASH programs. The other two courses with at least one menstruation focused class session concentrated on the biological aspects of menstruation and reproductive health. Several classes incorporated readings (*n* = 6), ranging in topics from the biological implications of the age of menarche, to a toolkit on how to integrate menstrual hygiene management into humanitarian response. Two courses incorporated assignments related to menstruation, focusing on the potential social and cultural impacts of menstruation on the lives of women and girls. Courses including menstrual hygiene or related topics that did not necessarily fall into a defined level of inclusion were categorized as “Other” (*n* = 12), and the majority of these were cross-listed courses.

**Table 2:** Level of Inclusion of menstruation-related topics in public health courses by area of specialization.

**Area of Specialization**	**Level of inclusion**						
	**Full course**	**Full module**	**Class Session**	**Reading**	**Assignment**	**Other**	**Total**
Behavioral and social science						2	2
Biostatistics and informatics							0
Community health			1			1	2
Environmental health		1	2			1	4
Epidemiology						2	2
Global health			1	1		2	4
Health policy and management							0
Health promotion and communication							0
Maternal and child health				1		2	3
Minority health and health disparities							0
Other				3	2	1	6
Cross-cutting			1	1		3	5
Total		1	5	6	2	14	28

## Discussion

This review provides important insights about the current inclusion of menstruation-related content in the curricula of the top 20 US schools of public health. Only 14 of the 20 schools (70%) had courses that included any content on menstruation-related issues, and in most cases this inclusion was nominal, such as a single reading or assignment. Between these 14 schools, only 28 courses included this content. Despite the small number of classes analyzed, some interesting patterns arose in terms of the type of information included and the types of courses in which this information was included. These patterns aligned with those seen within menstruation-related programming, policy, and research. Most frequently, these inclusions focused on menstruation and pubertal timing from a biological perspective, or on menstruation within the frame of water and sanitation. Although these aspects of menstruation are important, they represent a fraction of the range of menstruation-related issues that must be explored and addressed within population health. As the ways in which menstruation influences women and girls' lives throughout the lifecourse gain attention within public health domestically and globally, there is an urgent need to include curricular content on the complex and layered societal beliefs associated with it, and the range of implications of menstrual onset and menstruation for population health. This will in turn ensure graduates are well-equipped to tackle this issue.

Although courses that included menstruation topics ranged across area of specialization, content was most commonly found within global health, environmental health, and maternal and child health. The rationale for such inclusion is clear. To date research, policy and programming has focused on menstruation within low- and middle- income countries (global health), with interventions to address menstruation-related challenges disproportionately led by the WASH sector (environmental health). While there is a growing recognition that not all people who menstruate are female, almost all research, policy and programming to date has focused on women and girls (maternal and child health) (3, 16). Many of the courses including menstruation-related topics were cross-listed across area of specialization (*n* = 5) or were interdisciplinary (*n* = 6). One of the biggest strengths and challenges of menstruation is its interdisciplinarity; while it fits within multiple areas, it does not have one clear home within public health. This may in turn lead to it being inadvertently overlooked in relation to course content coverage.

Despite the current concentration of menstrual-related topics within a few select areas of specialization, menstruation is a cross-cutting issue, with relevancy for graduate level training across public health specializations (3). For instance, while the review found no courses with related content within Minority Health and Health Disparities, there are many important areas for exploration. For example, African-American girls experience menarche at a younger age than their White, Hispanic or Asian counterparts, and girls of low socioeconomic status often lack information, education and support about menarche and puberty (20, 24). Similarly, our review found no instances in which menstruation-related issues were discussed within Health Policy and Management. However, there are an increasing number of laws and policies related to the provision of menstrual products both domestically and globally, which will require critical analysis and evaluation of uptake and impact (26, 28). Notably, while our review found no courses within Health Promotion and Communication that included menstruation, there is a critical need for improved puberty education and menstrual health promotion within the US and globally (24, 31). It is possible that health promotion and communication content was included as part of the coursework for the areas of specialization with menstruation-related courses; however, it was not evident within the content of the reviewed courses.

Adding new content to ASPPH curricula is not a small task given the many competing topics and limited length of graduate-level public health training. However, the literature highlights a few key factors for successfully integrating new content into public health curricula, including sector recognition (32, 33), demonstrated student interest (34), and early adopters and champions. To date, menstruation-related topics have made some progress in relation to these facilitating factors. There is growing recognition from national and international advocacy, media and policy actors on the importance of this topic (*Sector recognition)*. Although there is no rigorous evidence indicating student interest, the authors' have anecdotally witnessed growing interest from students (*Student Interest)*. As this review has revealed, there are a small number of schools and professors who are addressing this subject matter in their coursework (*Early adopters)*. These early adopters can provide useful models and guidance on adapting curricula to better integrate this topic.

### Limitations

There were a number of limitations. One, only the top 20 accredited US public health schools were included, and thus coursework on menstruation in other public health schools or programs was likely missed. We chose to review the curricula of schools to capture the wider breadth of what a school might teach given the broader range of degree options typically offered at these institutions as compared to public health programs. We reviewed only 20 schools because the intention was to create a foundation for additional exploration. Two, the approach included a desk review and electronic outreach using a select number of search terms and topics, thus courses may have been missed that did not include these terms. In addition, 101 courses met the initial criteria for inclusion that were not analyzed because we were unable to obtain the syllabi.

### Recommendations

A key recommendation is for schools to review their existing curricula for menstruation-related health and hygiene content and application to public health competencies of relevance. This might include, e.g., designing programming or identifying key research questions and how to examine them within the spheres of adolescent health, women's health, reproductive health, health policy, environmental health, health education, and humanitarian response (17, 18, 31). Second, a more in-depth analysis of how menstruation-related topics have been incorporated into existing courses and disciplinary homes would provide more concrete guidance to schools seeking to build menstruation-related issues into their curricula.

## Conclusions

Although there exists growing global interest in “menstruation” as a public health issue, and a rapidly evolving menstrual policy landscape, menstruation-related content remains limited within graduate-level US public health curricula. Today's public health students thus may lack sufficient understanding of the many ways in which menstruation affects the lives of women, girls, and other people with periods, hindering the examination of critical research questions, or understanding of how to address it through improved programming and policy, both of which in turn are critical for assuring population health domestically and globally.

## Data Availability Statement

The raw data supporting the conclusions of this article will be made available by the authors, without undue reservation.

## Author Contributions

MS conceived the study, supported the analysis, and drafted the manuscript. CL and CG participated in data collection, analysis, and contributed to drafting the manuscript. DL participated in study conception and data collection. All authors read and approved the final manuscript.

## Conflict of Interest

The authors declare that the research was conducted in the absence of any commercial or financial relationships that could be construed as a potential conflict of interest.
